# Tetra-μ-acetato-κ^8^ *O*:*O*′-bis­{[4-methyl-*N*-(4-methyl­phen­yl)pyridin-2-amine-κ*N* ^1^]copper(II)}

**DOI:** 10.1107/S1600536810031168

**Published:** 2010-08-11

**Authors:** Zainal A. Fairuz, Zaharah Aiyub, Zanariah Abdullah, Seik Weng Ng, Edward R. T. Tiekink

**Affiliations:** a Department of Chemistry, University of Malaya, 50603 Kuala Lumpur, Malaysia

## Abstract

The title complex, [Cu_2_(CH_3_COO)_4_(C_13_H_14_N_2_)_2_], features a binuclear mol­ecule, which lies about a crystallographic centre of inversion; the four acetate ions each bridge a pair of Cu^II^ atoms. The coordination of the metal atom is distorted octa­hedral within a donor set defined by four O atoms, the heterocyclic N atom and the second Cu atom. The pyridine ring is twisted with respect to the tolyl ring and forms a dihedral angle of 35.34 (9)°. A bifurcated N—H⋯(O,O) hydrogen bond is present, linking the amine group to two carboxyl­ate O atoms derived from different acetate ions. In the crystal, C—H⋯π inter­actions link mol­ecules into a supra­molecular array in the *bc* plane.

## Related literature

For examples of tetra­kis­acetato­bis­[(substituted 2-amino­pyrid­yl)copper(II) complexes, see: Barquín *et al.* (2004 ▶); Seco *et al.* (2004 ▶); Sieroń (2004 ▶); Fairuz *et al.* (2010 ▶).
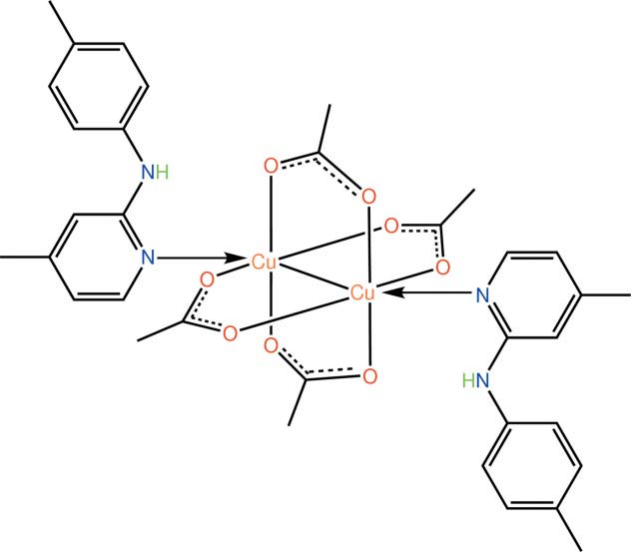


## Experimental

### 

#### Crystal data

[Cu_2_(C_2_H_3_O_2_)_4_(C_13_H_14_N_2_)_2_]
*M* *_r_* = 759.78
Monoclinic, 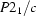

*a* = 11.7519 (6) Å
*b* = 15.5822 (8) Å
*c* = 9.9050 (5) Å
β = 110.5698 (6)°
*V* = 1698.17 (15) Å^3^
*Z* = 2
Mo *K*α radiation
μ = 1.31 mm^−1^
*T* = 293 K
0.40 × 0.20 × 0.10 mm

#### Data collection

Bruker SMART APEX CCD diffractometer
Absorption correction: multi-scan (*SADABS*; Sheldrick, 1996 ▶) *T*_min_ = 0.648, *T*_max_ = 0.746
16009 measured reflections
3890 independent reflections
3462 reflections with *I* > 2σ(*I*)
*R*_int_ = 0.019

#### Refinement

*R*[*F* ^2^ > 2σ(*F* ^2^)] = 0.027
*wR*(*F* ^2^) = 0.083
*S* = 1.01
3890 reflections
225 parameters
1 restraint
H atoms treated by a mixture of independent and constrained refinement
Δρ_max_ = 0.32 e Å^−3^
Δρ_min_ = −0.26 e Å^−3^

### 

Data collection: *APEX2* (Bruker, 2009 ▶); cell refinement: *SAINT* (Bruker, 2009 ▶); data reduction: *SAINT*; program(s) used to solve structure: *SHELXS97* (Sheldrick, 2008 ▶); program(s) used to refine structure: *SHELXL97* (Sheldrick, 2008 ▶); molecular graphics: *ORTEP-3* (Farrugia, 1997 ▶) and *DIAMOND* (Brandenburg, 2006 ▶); software used to prepare material for publication: *publCIF* (Westrip, 2010 ▶).

## Supplementary Material

Crystal structure: contains datablocks global, I. DOI: 10.1107/S1600536810031168/hb5591sup1.cif

Structure factors: contains datablocks I. DOI: 10.1107/S1600536810031168/hb5591Isup2.hkl

Additional supplementary materials:  crystallographic information; 3D view; checkCIF report

## Figures and Tables

**Table 1:** Selected bond lengths (Å)

Cu1—O2^i^	1.9701 (13)
Cu1—O3	1.9702 (14)
Cu1—O4^i^	1.9713 (14)
Cu1—O1	1.9759 (13)
Cu1—N1	2.2016 (14)
Cu1—Cu1^i^	2.6480 (4)

Symmetry code: (i) 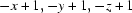.

**Table 2:** Hydrogen-bond geometry (Å, °) *Cg*1 is the centroid of the N1,C5–C9 ring.

*D*—H⋯*A*	*D*—H	H⋯*A*	*D*⋯*A*	*D*—H⋯*A*
N2—H2*n*⋯O1	0.85 (3)	2.36 (2)	3.117 (3)	149 (2)
N2—H2*n*⋯O4^i^	0.85 (3)	2.46 (3)	3.047 (2)	127 (2)
C2—H2a⋯*Cg*1^ii^	0.96	2.80	3.566 (2)	138

Symmetry codes: (i) 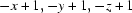; (ii) 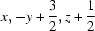.

**Fig. 1.**
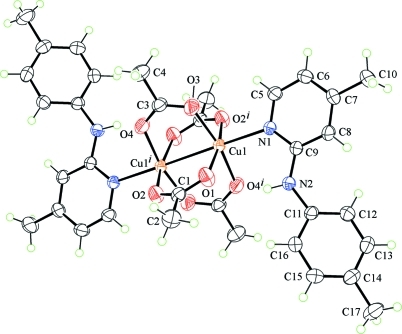
The molecular structure of (I) showing displacement ellipsoids at the 50% probability level. Symmetry code: (i) 1 - x, 1 - y, 1 - z.

**Fig. 2.**
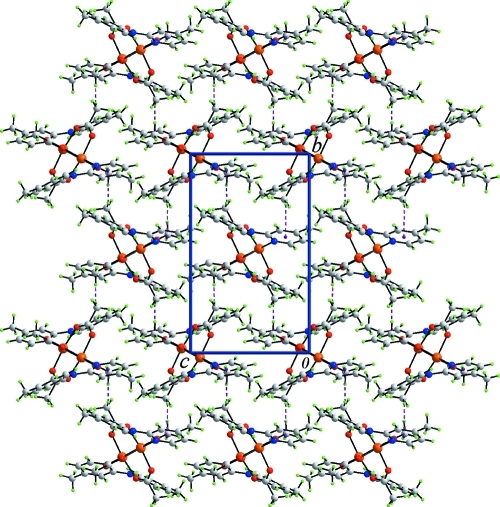
Supramolecular arrays in the bc plane in (I) mediated by C–H···π interactions shown as purple dashed lines.

**Fig. 3.**
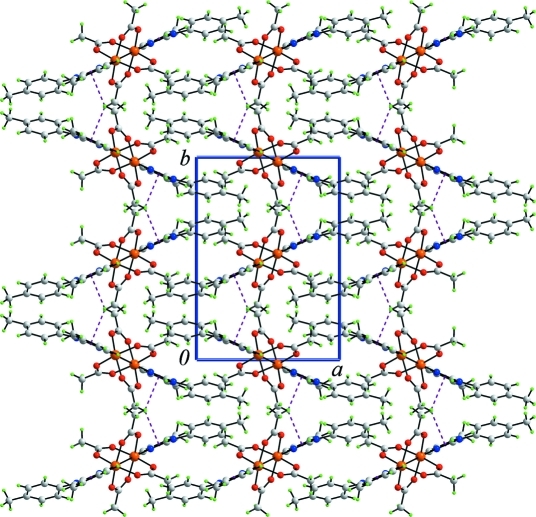
Unit-cell contents shown in projection down the c axis in (I) showing the stacking of layers along a. The C–H···π contacts are shown as purple dashed lines.

## supplementary crystallographic information

### Comment

In connection with on-going studies into the structural characterization of tetrakisacetatobis[(substituted 2-aminopyridyl)copper] complexes, see: Barquín *et al.*, 2004; Seco *et al.*, 2004; Sieroń, 2004; Fairuz *et al.*, 2010), the binuclear title complex, (I), was investigated.

The binuclear copper(II) complex, Fig. 1, is situated about a centre of inversion and features two Cu^II^ atoms bridged by four acetate groups. The Cu–O bond distances lie in a narrow range, *i.e.* 1.9701 (13) to 1.9759 (13) Å, Table 1. The distorted octahedral coordination environment for the Cu atom is completed by a pyridine-N atom derived from the 4-methyl-*N*-*p*-tolylpyridin-2-amine ligand and the second Cu atom [Cu···Cu^i^ = 2.6480 (4) Å for *i*: 1 - *x*, 1 - *y*, 1 - *z*]. Two intramolecular N1–H···O interactions are noted in which the amine-H spans carboxylate-O atoms derived from different ligands, Table 2. The dihedral angle formed between the pyridine and benzene rings of 35.34 (9) ° indicates the *N*-heterocycle is non-planar. The major twist in the molecule occurs around the amine group as seen in the value of the C9–N2–C11–C12 torsion angle of -27.2 (3) °. In the crystal packing, contacts of the type C–H···π occur between methyl-H and pyridine rings, Table 2, and lead to the formation of supramolecular arrays in the *bc* plane, Fig. 2. Layers thus formed stack along the *a* axis, Fig. 3.

### Experimental

Copper acetate (0.1 g, 0.5 mmol) dissolved in acetonitrile (15 ml) was added to a mixture of 4-methyl-*N*-*p*-tolylpyridin-2-amine (0.2183 g, 1.1 mmol) and trimethyl orthoformate (10 ml). The mixture was heated at 323 K, the green precipitate was collected and recrystallization from its acetonitrile solution yielded green prisms of (I).

### Refinement

Carbon-bound H-atoms were placed in calculated positions (C—H 0.93 to 0.96 Å) and were included in the refinement in the riding model approximation, with *U*_iso_(H) set to 1.2 to 1.5*U*_equiv_(C). The N-bound H-atom was located in a difference Fourier map, and was refined with a distance restraint of N–H 0.86±0.01 Å; the *U_iso_* value was freely refined

### Crystal data

**Table:**

[Cu_2_(C_2_H_3_O_2_)_4_(C_13_H_14_N_2_)_2_]	*F*(000) = 788
*M**_r_* = 759.78	*D*_x_ = 1.486 Mg m^−^^3^
Monoclinic, *P*2_1_/*c*	Mo *K*α radiation, λ = 0.71073 Å
Hall symbol: -P 2ybc	Cell parameters from 8249 reflections
*a* = 11.7519 (6) Å	θ = 4.3–28.2°
*b* = 15.5822 (8) Å	µ = 1.31 mm^−^^1^
*c* = 9.9050 (5) Å	*T* = 293 K
β = 110.5698 (6)°	Prism, green
*V* = 1698.17 (15) Å^3^	0.40 × 0.20 × 0.10 mm
*Z* = 2	

### Data collection

**Table:**

Bruker SMART APEX CCD diffractometer	3890 independent reflections
Radiation source: fine-focus sealed tube	3462 reflections with *I* > 2σ(*I*)
graphite	*R*_int_ = 0.019
ω scan	θ_max_ = 27.5°, θ_min_ = 1.9°
Absorption correction: multi-scan (*SADABS*; Sheldrick, 1996)	*h* = −14→15
*T*_min_ = 0.648, *T*_max_ = 0.746	*k* = −20→19
16009 measured reflections	*l* = −12→12

### Refinement

**Table:**

Refinement on *F*^2^	Primary atom site location: structure-invariant direct methods
Least-squares matrix: full	Secondary atom site location: difference Fourier map
*R*[*F*^2^ > 2σ(*F*^2^)] = 0.027	Hydrogen site location: inferred from neighbouring sites
*wR*(*F*^2^) = 0.083	H atoms treated by a mixture of independent and constrained refinement
*S* = 1.01	*w* = 1/[σ^2^(*F*_o_^2^) + (0.0493*P*)^2^ + 0.7683*P*] where *P* = (*F*_o_^2^ + 2*F*_c_^2^)/3
3890 reflections	(Δ/σ)_max_ = 0.001
225 parameters	Δρ_max_ = 0.32 e Å^−^^3^
1 restraint	Δρ_min_ = −0.26 e Å^−^^3^

### Special details

**Table:**

Geometry. All e.s.d.'s (except the e.s.d. in the dihedral angle between two l.s. planes) are estimated using the full covariance matrix. The cell e.s.d.'s are taken into account individually in the estimation of e.s.d.'s in distances, angles and torsion angles; correlations between e.s.d.'s in cell parameters are only used when they are defined by crystal symmetry. An approximate (isotropic) treatment of cell e.s.d.'s is used for estimating e.s.d.'s involving l.s. planes.
Refinement. Refinement of *F*^2^ against ALL reflections. The weighted *R*-factor *wR* and goodness of fit *S* are based on *F*^2^, conventional *R*-factors *R* are based on *F*, with *F* set to zero for negative *F*^2^. The threshold expression of *F*^2^ > σ(*F*^2^) is used only for calculating *R*-factors(gt) *etc*. and is not relevant to the choice of reflections for refinement. *R*-factors based on *F*^2^ are statistically about twice as large as those based on *F*, and *R*- factors based on ALL data will be even larger.

### Fractional atomic coordinates and isotropic or equivalent isotropic displacement parameters (Å^2^)

**Table:**

	*x*	*y*	*z*	*U*_iso_*/*U*_eq_	
Cu1	0.566326 (17)	0.523188 (13)	0.42225 (2)	0.02759 (8)	
N1	0.67899 (13)	0.56104 (9)	0.29545 (15)	0.0288 (3)	
N2	0.83831 (15)	0.60834 (13)	0.48943 (17)	0.0431 (4)	
H2n	0.7883 (19)	0.6036 (17)	0.533 (3)	0.060 (8)*	
O1	0.59163 (14)	0.62952 (9)	0.53779 (16)	0.0456 (3)	
O2	0.47897 (13)	0.59061 (9)	0.66720 (15)	0.0416 (3)	
O3	0.41276 (12)	0.56868 (10)	0.28515 (15)	0.0449 (3)	
O4	0.30200 (12)	0.52953 (10)	0.41638 (16)	0.0444 (3)	
C1	0.54512 (15)	0.64284 (11)	0.63233 (19)	0.0319 (4)	
C2	0.5712 (2)	0.72788 (14)	0.7088 (3)	0.0476 (5)	
H2A	0.6411	0.7535	0.6962	0.071*	
H2B	0.5870	0.7194	0.8098	0.071*	
H2C	0.5023	0.7651	0.6694	0.071*	
C3	0.31446 (16)	0.56196 (12)	0.30683 (19)	0.0343 (4)	
C4	0.2011 (2)	0.59515 (18)	0.1922 (3)	0.0570 (6)	
H4A	0.1309	0.5714	0.2067	0.086*	
H4B	0.2013	0.5786	0.0989	0.086*	
H4C	0.1989	0.6566	0.1979	0.086*	
C5	0.62691 (17)	0.54835 (13)	0.1533 (2)	0.0376 (4)	
H5	0.5491	0.5250	0.1193	0.045*	
C6	0.68042 (18)	0.56738 (15)	0.0545 (2)	0.0426 (5)	
H6	0.6405	0.5564	−0.0431	0.051*	
C7	0.79636 (18)	0.60370 (13)	0.1037 (2)	0.0381 (4)	
C8	0.85144 (16)	0.61737 (12)	0.24918 (19)	0.0342 (4)	
H8	0.9286	0.6417	0.2848	0.041*	
C9	0.79178 (15)	0.59485 (11)	0.34396 (18)	0.0287 (3)	
C10	0.8574 (2)	0.6289 (2)	−0.0006 (2)	0.0594 (7)	
H10A	0.9330	0.5986	0.0225	0.089*	
H10B	0.8725	0.6895	0.0059	0.089*	
H10C	0.8056	0.6146	−0.0969	0.089*	
C11	0.95658 (16)	0.62975 (12)	0.58042 (17)	0.0313 (4)	
C12	1.06199 (17)	0.60942 (12)	0.5541 (2)	0.0351 (4)	
H12	1.0573	0.5808	0.4700	0.042*	
C13	1.17443 (17)	0.63203 (13)	0.6539 (2)	0.0383 (4)	
H13	1.2441	0.6197	0.6335	0.046*	
C14	1.18612 (17)	0.67236 (13)	0.7825 (2)	0.0391 (4)	
C15	1.07993 (18)	0.69041 (14)	0.8091 (2)	0.0422 (4)	
H15	1.0848	0.7165	0.8954	0.051*	
C16	0.96734 (17)	0.67014 (14)	0.7092 (2)	0.0382 (4)	
H16	0.8976	0.6838	0.7286	0.046*	
C17	1.3088 (2)	0.69482 (19)	0.8908 (3)	0.0605 (6)	
H17A	1.3591	0.7183	0.8416	0.091*	
H17B	1.3464	0.6441	0.9423	0.091*	
H17C	1.2993	0.7364	0.9575	0.091*	

### Atomic displacement parameters (Å^2^)

**Table:**

	*U*^11^	*U*^22^	*U*^33^	*U*^12^	*U*^13^	*U*^23^
Cu1	0.02400 (12)	0.03200 (13)	0.02806 (12)	−0.00382 (8)	0.01077 (8)	−0.00001 (7)
N1	0.0256 (7)	0.0335 (7)	0.0283 (7)	−0.0050 (6)	0.0107 (5)	−0.0009 (6)
N2	0.0290 (8)	0.0746 (12)	0.0281 (7)	−0.0196 (8)	0.0129 (6)	−0.0065 (7)
O1	0.0547 (9)	0.0384 (7)	0.0547 (8)	−0.0154 (6)	0.0331 (7)	−0.0131 (6)
O2	0.0451 (8)	0.0390 (7)	0.0475 (8)	−0.0118 (6)	0.0248 (6)	−0.0106 (6)
O3	0.0342 (7)	0.0612 (9)	0.0396 (7)	0.0082 (7)	0.0133 (6)	0.0156 (7)
O4	0.0285 (7)	0.0626 (10)	0.0407 (7)	0.0018 (6)	0.0102 (6)	0.0122 (6)
C1	0.0273 (8)	0.0305 (8)	0.0353 (9)	−0.0009 (7)	0.0076 (7)	−0.0028 (7)
C2	0.0500 (12)	0.0361 (10)	0.0557 (12)	−0.0078 (9)	0.0172 (10)	−0.0128 (9)
C3	0.0299 (9)	0.0369 (9)	0.0320 (8)	0.0046 (7)	0.0057 (7)	0.0001 (7)
C4	0.0399 (11)	0.0736 (16)	0.0467 (12)	0.0173 (11)	0.0017 (9)	0.0116 (11)
C5	0.0312 (9)	0.0492 (10)	0.0314 (9)	−0.0133 (8)	0.0097 (7)	−0.0049 (8)
C6	0.0404 (10)	0.0600 (13)	0.0270 (8)	−0.0120 (9)	0.0114 (7)	−0.0047 (8)
C7	0.0368 (9)	0.0497 (11)	0.0326 (9)	−0.0042 (8)	0.0183 (8)	0.0027 (8)
C8	0.0265 (8)	0.0438 (10)	0.0334 (8)	−0.0072 (7)	0.0120 (7)	0.0038 (7)
C9	0.0261 (8)	0.0316 (8)	0.0291 (8)	−0.0032 (6)	0.0107 (6)	−0.0002 (6)
C10	0.0529 (13)	0.0952 (19)	0.0381 (11)	−0.0155 (13)	0.0260 (10)	0.0041 (12)
C11	0.0277 (8)	0.0370 (9)	0.0283 (8)	−0.0073 (7)	0.0086 (6)	0.0016 (6)
C12	0.0355 (9)	0.0372 (9)	0.0335 (9)	−0.0017 (7)	0.0133 (7)	−0.0034 (7)
C13	0.0276 (8)	0.0427 (10)	0.0445 (10)	0.0012 (8)	0.0125 (8)	0.0025 (8)
C14	0.0301 (9)	0.0421 (10)	0.0386 (9)	−0.0039 (8)	0.0041 (7)	0.0025 (8)
C15	0.0406 (10)	0.0513 (11)	0.0316 (9)	−0.0028 (9)	0.0090 (8)	−0.0080 (8)
C16	0.0299 (9)	0.0535 (11)	0.0333 (9)	−0.0021 (8)	0.0135 (7)	−0.0029 (8)
C17	0.0357 (11)	0.0785 (17)	0.0535 (13)	−0.0105 (11)	−0.0015 (10)	−0.0050 (12)

### Geometric parameters (Å, °)

**Table:**

Cu1—O2^i^	1.9701 (13)	C5—C6	1.368 (3)
Cu1—O3	1.9702 (14)	C5—H5	0.9300
Cu1—O4^i^	1.9713 (14)	C6—C7	1.396 (3)
Cu1—O1	1.9759 (13)	C6—H6	0.9300
Cu1—N1	2.2016 (14)	C7—C8	1.373 (3)
Cu1—Cu1^i^	2.6480 (4)	C7—C10	1.502 (3)
N1—C5	1.339 (2)	C8—C9	1.400 (2)
N1—C9	1.348 (2)	C8—H8	0.9300
N2—C9	1.366 (2)	C10—H10A	0.9600
N2—C11	1.405 (2)	C10—H10B	0.9600
N2—H2n	0.85 (3)	C10—H10C	0.9600
O1—C1	1.256 (2)	C11—C16	1.388 (2)
O2—C1	1.255 (2)	C11—C12	1.389 (3)
O2—Cu1^i^	1.9701 (13)	C12—C13	1.389 (3)
O3—C3	1.251 (2)	C12—H12	0.9300
O4—C3	1.251 (2)	C13—C14	1.384 (3)
O4—Cu1^i^	1.9713 (14)	C13—H13	0.9300
C1—C2	1.504 (3)	C14—C15	1.391 (3)
C2—H2A	0.9600	C14—C17	1.505 (3)
C2—H2B	0.9600	C15—C16	1.382 (3)
C2—H2C	0.9600	C15—H15	0.9300
C3—C4	1.506 (3)	C16—H16	0.9300
C4—H4A	0.9600	C17—H17A	0.9600
C4—H4B	0.9600	C17—H17B	0.9600
C4—H4C	0.9600	C17—H17C	0.9600
			
O2^i^—Cu1—O3	88.66 (7)	N1—C5—H5	117.8
O2^i^—Cu1—O4^i^	90.04 (7)	C6—C5—H5	117.8
O3—Cu1—O4^i^	167.64 (6)	C5—C6—C7	118.40 (17)
O2^i^—Cu1—O1	167.72 (6)	C5—C6—H6	120.8
O3—Cu1—O1	90.72 (7)	C7—C6—H6	120.8
O4^i^—Cu1—O1	87.95 (7)	C8—C7—C6	118.17 (16)
O2^i^—Cu1—N1	96.10 (5)	C8—C7—C10	121.22 (18)
O3—Cu1—N1	95.70 (5)	C6—C7—C10	120.60 (18)
O4^i^—Cu1—N1	96.67 (6)	C7—C8—C9	120.16 (16)
O1—Cu1—N1	96.17 (5)	C7—C8—H8	119.9
O2^i^—Cu1—Cu1^i^	83.96 (4)	C9—C8—H8	119.9
O3—Cu1—Cu1^i^	85.14 (4)	N1—C9—N2	114.53 (14)
O4^i^—Cu1—Cu1^i^	82.50 (4)	N1—C9—C8	121.36 (15)
O1—Cu1—Cu1^i^	83.77 (4)	N2—C9—C8	124.04 (16)
N1—Cu1—Cu1^i^	179.17 (4)	C7—C10—H10A	109.5
C5—N1—C9	117.49 (14)	C7—C10—H10B	109.5
C5—N1—Cu1	114.60 (11)	H10A—C10—H10B	109.5
C9—N1—Cu1	127.91 (11)	C7—C10—H10C	109.5
C9—N2—C11	130.71 (15)	H10A—C10—H10C	109.5
C9—N2—H2n	115.7 (18)	H10B—C10—H10C	109.5
C11—N2—H2n	113.6 (18)	C16—C11—C12	118.46 (16)
C1—O1—Cu1	123.64 (12)	C16—C11—N2	116.57 (16)
C1—O2—Cu1^i^	123.75 (12)	C12—C11—N2	124.87 (16)
C3—O3—Cu1	121.93 (12)	C13—C12—C11	119.80 (17)
C3—O4—Cu1^i^	125.01 (12)	C13—C12—H12	120.1
O1—C1—O2	124.88 (16)	C11—C12—H12	120.1
O1—C1—C2	117.13 (17)	C12—C13—C14	122.15 (18)
O2—C1—C2	117.99 (17)	C12—C13—H13	118.9
C1—C2—H2A	109.5	C14—C13—H13	118.9
C1—C2—H2B	109.5	C13—C14—C15	117.41 (17)
H2A—C2—H2B	109.5	C13—C14—C17	121.47 (19)
C1—C2—H2C	109.5	C15—C14—C17	121.1 (2)
H2A—C2—H2C	109.5	C16—C15—C14	121.02 (18)
H2B—C2—H2C	109.5	C16—C15—H15	119.5
O4—C3—O3	125.38 (17)	C14—C15—H15	119.5
O4—C3—C4	116.79 (18)	C15—C16—C11	121.12 (18)
O3—C3—C4	117.83 (18)	C15—C16—H16	119.4
C3—C4—H4A	109.5	C11—C16—H16	119.4
C3—C4—H4B	109.5	C14—C17—H17A	109.5
H4A—C4—H4B	109.5	C14—C17—H17B	109.5
C3—C4—H4C	109.5	H17A—C17—H17B	109.5
H4A—C4—H4C	109.5	C14—C17—H17C	109.5
H4B—C4—H4C	109.5	H17A—C17—H17C	109.5
N1—C5—C6	124.41 (17)	H17B—C17—H17C	109.5
			
O2^i^—Cu1—N1—C5	48.27 (14)	C9—N1—C5—C6	0.0 (3)
O3—Cu1—N1—C5	−40.99 (15)	Cu1—N1—C5—C6	179.90 (18)
O4^i^—Cu1—N1—C5	139.02 (14)	N1—C5—C6—C7	−1.0 (3)
O1—Cu1—N1—C5	−132.33 (14)	C5—C6—C7—C8	0.9 (3)
Cu1^i^—Cu1—N1—C5	142 (3)	C5—C6—C7—C10	−177.7 (2)
O2^i^—Cu1—N1—C9	−131.83 (15)	C6—C7—C8—C9	0.2 (3)
O3—Cu1—N1—C9	138.92 (15)	C10—C7—C8—C9	178.8 (2)
O4^i^—Cu1—N1—C9	−41.07 (15)	C5—N1—C9—N2	178.27 (18)
O1—Cu1—N1—C9	47.58 (15)	Cu1—N1—C9—N2	−1.6 (2)
Cu1^i^—Cu1—N1—C9	−38 (3)	C5—N1—C9—C8	1.2 (3)
O2^i^—Cu1—O1—C1	−2.9 (4)	Cu1—N1—C9—C8	−178.74 (13)
O3—Cu1—O1—C1	84.09 (16)	C11—N2—C9—N1	169.2 (2)
O4^i^—Cu1—O1—C1	−83.62 (16)	C11—N2—C9—C8	−13.8 (3)
N1—Cu1—O1—C1	179.90 (16)	C7—C8—C9—N1	−1.3 (3)
Cu1^i^—Cu1—O1—C1	−0.94 (15)	C7—C8—C9—N2	−178.1 (2)
O2^i^—Cu1—O3—C3	82.85 (16)	C9—N2—C11—C16	156.4 (2)
O4^i^—Cu1—O3—C3	−1.2 (4)	C9—N2—C11—C12	−27.2 (3)
O1—Cu1—O3—C3	−84.89 (16)	C16—C11—C12—C13	−2.0 (3)
N1—Cu1—O3—C3	178.84 (16)	N2—C11—C12—C13	−178.34 (18)
Cu1^i^—Cu1—O3—C3	−1.20 (15)	C11—C12—C13—C14	2.0 (3)
Cu1—O1—C1—O2	1.2 (3)	C12—C13—C14—C15	−0.4 (3)
Cu1—O1—C1—C2	−178.81 (14)	C12—C13—C14—C17	178.7 (2)
Cu1^i^—O2—C1—O1	−0.6 (3)	C13—C14—C15—C16	−1.2 (3)
Cu1^i^—O2—C1—C2	179.40 (14)	C17—C14—C15—C16	179.7 (2)
Cu1^i^—O4—C3—O3	−2.7 (3)	C14—C15—C16—C11	1.2 (3)
Cu1^i^—O4—C3—C4	177.10 (15)	C12—C11—C16—C15	0.4 (3)
Cu1—O3—C3—O4	2.6 (3)	N2—C11—C16—C15	177.08 (19)
Cu1—O3—C3—C4	−177.18 (15)		

Symmetry codes: (i) −*x*+1, −*y*+1, −*z*+1.

### Hydrogen-bond geometry (Å, °)

**Table:**

Cg1 is the centroid of the N1,C5–C9 ring.

**Table:**

*D*—H···*A*	*D*—H	H···*A*	*D*···*A*	*D*—H···*A*
N2—H2n···O1	0.85 (3)	2.36 (2)	3.117 (3)	149 (2)
N2—H2n···O4^i^	0.85 (3)	2.46 (3)	3.047 (2)	127 (2)
C2—H2a···Cg1^ii^	0.96	2.80	3.566 (2)	138

Symmetry codes:  (i) −*x*+1, −*y*+1, −*z*+1; (ii) *x*, −*y*+3/2, *z*+1/2.
